# Electro-acupuncture regulates glucose metabolism in chronic stress model rats

**DOI:** 10.1038/s41598-020-68132-w

**Published:** 2020-07-09

**Authors:** Fu-qiang Ma, Chan-juan Sun, Jun-jie Wei, Ya-dong Wang, Jia-cheng Shen, Jin-jian Chang

**Affiliations:** 1 Luoyang Traditional Chinese Medicine Hospital, Luoyang, 471000 People’s Republic of China; 2 Department of Traditional Chinese Medicine, Xiamen University, Xiamen, 361005 People’s Republic of China; 3 Shanghai Research Institute of Acupuncture and Meridian, Shanghai, 200030 People’s Republic of China

**Keywords:** Molecular biology, Neuroscience, Physiology

## Abstract

Studies have shown that acupuncture is very effective in treating chronic stress depression. However, little is known about the therapeutic mechanism of electro-acupuncture. Metabolomics, on the other hand, is a technology that determines the metabolic changes of organisms caused by various interventions as a whole and is related to the overall effect of electro-acupuncture (EA). ^1^HNMR, serum sample analysis, and histopathology and molecular biology analysis were used to evaluate the effects of EA. The results show that electro-acupuncture points can regulate the heat pain threshold of chronic stress model rats and change the morphology of adrenal cortex cells Structure, and regulate the contents of corticotropin-releasing hormone, Corticosterone (CORT), glucose, alanine and valine in the samples. These findings help to clarify the therapeutic mechanism of electro-acupuncture on heterologous chronic stress model rats. The effect of electro-acupuncture on improving chronic stress is likely to be achieved by regulating glucose metabolism, which can provide a reference for clinical acupuncture treatment of chronic stress depression.

## Introduction

With the rapid development of the economy and society, everyone faces a stressful environment in daily life. Stress is a systemic, non-specific, adaptive response to any physiological or psychological stimulus that disrupts persistent homeostasis^1^. The effect of stress on the body seems to depend on the duration, not the intensity of external or internal stress stimuli^2^. The introduction of unpredictable factors makes it difficult for individuals to adapt to stressors. Therefore, chronic stress may induce and aggravate damage to tissues and organs^3^. Chronic stress is an important risk factor for depression^4^. Despite some findings about chronic stress, we still lack effective clinical treatments.

Acupuncture is the most popular adjuvant and alternative therapy in China, and it has been used for thousands of years^5^. Electro-acupuncture is an innovation of traditional Chinese acupuncture, which improves the clinical effect by transmitting electrical pulses to the needle^6^. Studies show that acupuncture has been used as an alternative therapy for depression in clinical practice^7–9^. Acupuncture can help reduce chronic stress-like behaviour by regulating hypothalamic–pituitary–adrenal (HPA) axis function^10^. However, it cannot reflect the biological state and regulatory function of the entire human body after external stimulation. At present, there are few studies on chronic stress-induced dysfunction in EA, and empirical evidence is lacking. Therefore, it is important to study the effects of EA on the mechanism of chronic stress effects.

Metabolomics can simultaneously monitor and evaluate changes in the metabolic spectrum caused by changes in disease and other stimuli in a holistic context. It can be characterised in a timely and sensitive manner^11,12^. This provides a good technical means for us to study the mechanism of chronic stress and the intervention effect of electro-acupuncture. Among them, ^1^H-NMR has the advantage of collecting non-destructive and non-selective information on samples^13^, which is one of the most commonly used methods in metabolomics. Its latest application shows the value of detecting biomarkers. It has a major impact on the discovery or diagnosis of a disease^14^. In our previous study, our research team used molecular biology and ^1^H-NMR techniques to evaluate the therapeutic effects and mechanisms of action of various diseases as a whole^15–17^. The chronic stress model has similar clinical characteristics to the human depression model and is widely used as an animal model of depression. The heterotypic intermittent stress (HIS) model was selected because of its unpredictable factors that make it difficult for rats to adapt to stressors.

In this study, a series of parameters were tested through ^1^HNMR-based metabolomics combined with pathological evaluation and molecular biology analysis, including heat pain threshold, adrenal pathology, adrenocortical hormone (ACTH), and corticosterone (CORT), glucose, alanine, and valine levels, and explore the potential mechanism of electro-acupuncture to improve glucose metabolism in heterogeneous intermittent stress (HIS) rats.

## Results

### Rat body weight and behavioural changes

**Figure 1:**
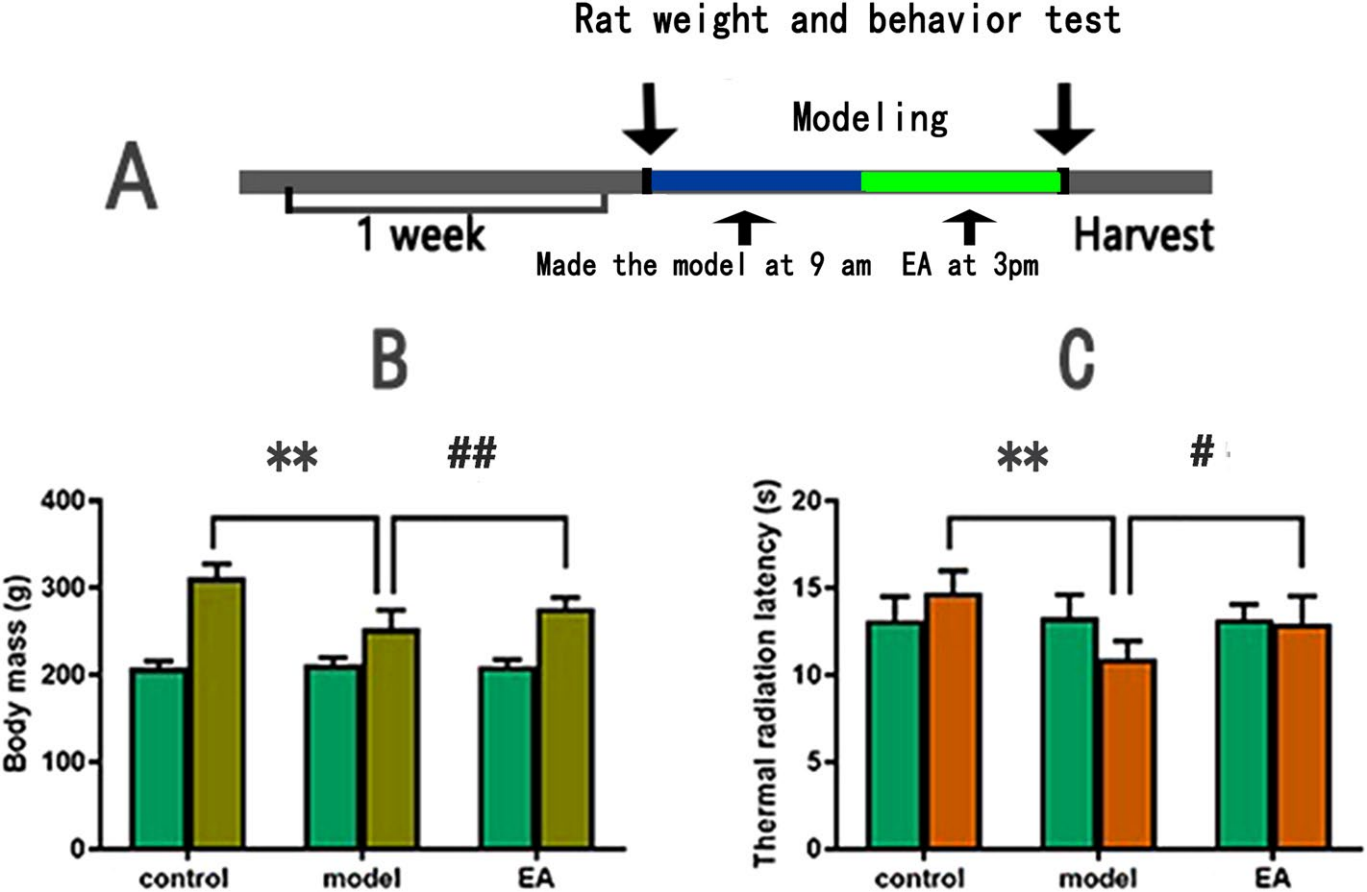
(**A**) Experimental protocol: all rats were fed adaptively for 7 days and randomly divided into three groups: control group (n = 25), model group (n = 25) and EA group (n = 25). Chronic stress models were made in model group and EA group. The 2 groups made models at 9 a.m., and the electro-acupuncture group started intervention at 3 p.m. The rats in the control group and model group were bound with 30 min with a rat retainer during electro-acupuncture. At baseline and 9 days later, the rats in each group were tested for body weight and thermal radiation latency, then anesthesia was induced with 4% isoflurane at 0.8 L air flow, then maintained with 1.5% isoflurane. Finally, the rats were killed and their serum, hypothalamus and adrenal tissues were detected. (**B**) Comparison of body weight of rats in each group. (**C**) Thermal radiation latency test of the soles of rats in each group (25 rats in each group experiment in (**B**) and (**C**)). Values are group mean ± SE. **p < 0.01 compared with control group; #*p* < 0.05; ##*p* < 0.01 versus model group. Based on one-way analysis of variance, multiple comparisons were performed by LSD test.

As illustrated in Fig. 1, there were no significant differences between the groups before the experiment, which were comparable (*p* > 0.05). However, a significant difference in body weight and thermal pain threshold was observed between groups following 9 days of HIS. The body weight and thermal pain threshold significantly decreased in the model group compared with the control group (*p* < 0.05). Compared with the model group, the electro-acupuncture group weight and thermal pain threshold were significantly increased (*p* < 0.05).

### Histological morphology examinations

**Figure 2:**
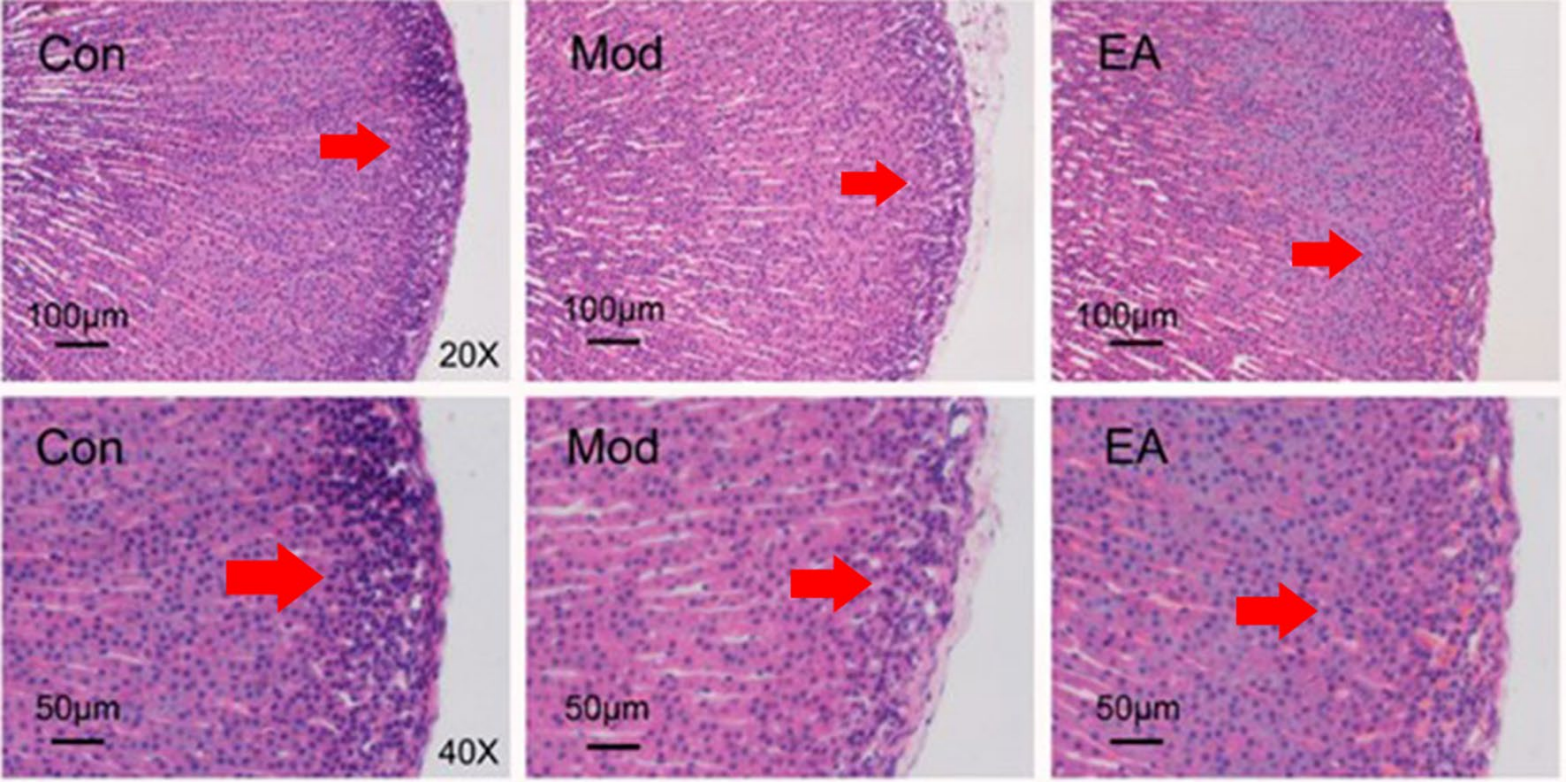
Histological examination of the adrenal glands. The 3 groups are the control group, the model group and the electro-acupuncture group. The upper row of the scale represents 100 µm, and the lower row represents 50 µm.

The histological morphology of adrenal tissues of rats in the 3 groups was observed under the microscope (in Fig. 2). The intact histological structure of adrenal tissue showed that the adrenal cortex can be divided into 3 parts: the zona glomerulosa, the zona fasciculata and the zona seticularis from the outside to the inside. Compared with the control group, the adrenal cortical zona glomerulosa cells in the model group were irregularly arranged, the nucleus was significantly reduced and the zona glomerulosa was thinner than the Control group, which shows that HIS rat modelling was well replicated. Compared with the HIS group, the spheroidal thickness and cell morphology of the EA group were improved to different degrees. This indicates that electro-acupuncture ST36 has a good effect on HIS rats.

### Examinations by enzyme-linked immunosorbent assay (ELISA)

**Figure 3:**
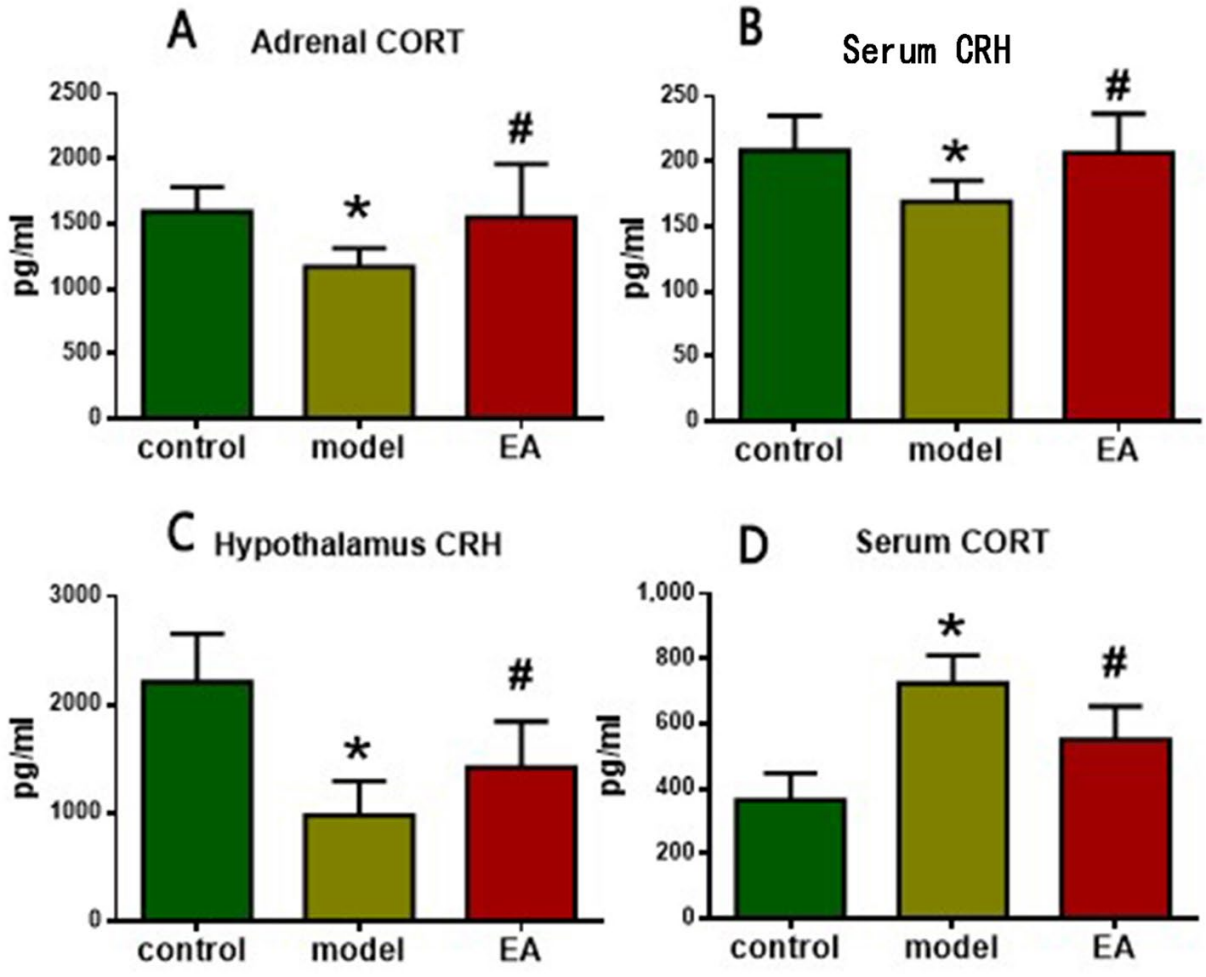
Expression of serum, hypothalamus and adrenal glands in 3 groups of rats. Nine rats from each group were randomly selected for testing (*means a statistical significance *p* < 0.05 when compared with the control group; #means a statistical significance *p* < 0.05 when compared with the model group) Based on one-way analysis of variance, multiple comparisons were performed by LSD test.

In this study, serum, hypothalamus and adrenal glands of all rats were tested by ELISA. The expressions of adrenal CORT, hypothalamus corticotropin-releasing hormone (CRH) and serum CRH in model group rats were lower than those in control group (*p* < 0.05), but the serum CORT levels were increased very much (*p* < 0.05). On the other hand, after electro-acupuncture treatment, 3 factors have increased significantly (*p* < 0.05); however, the serum CORT levels were extremely decreased (*p* < 0.05) (in Fig. 3).

### ^1^H NMR profiles of serum from rats

**Figure 4:**
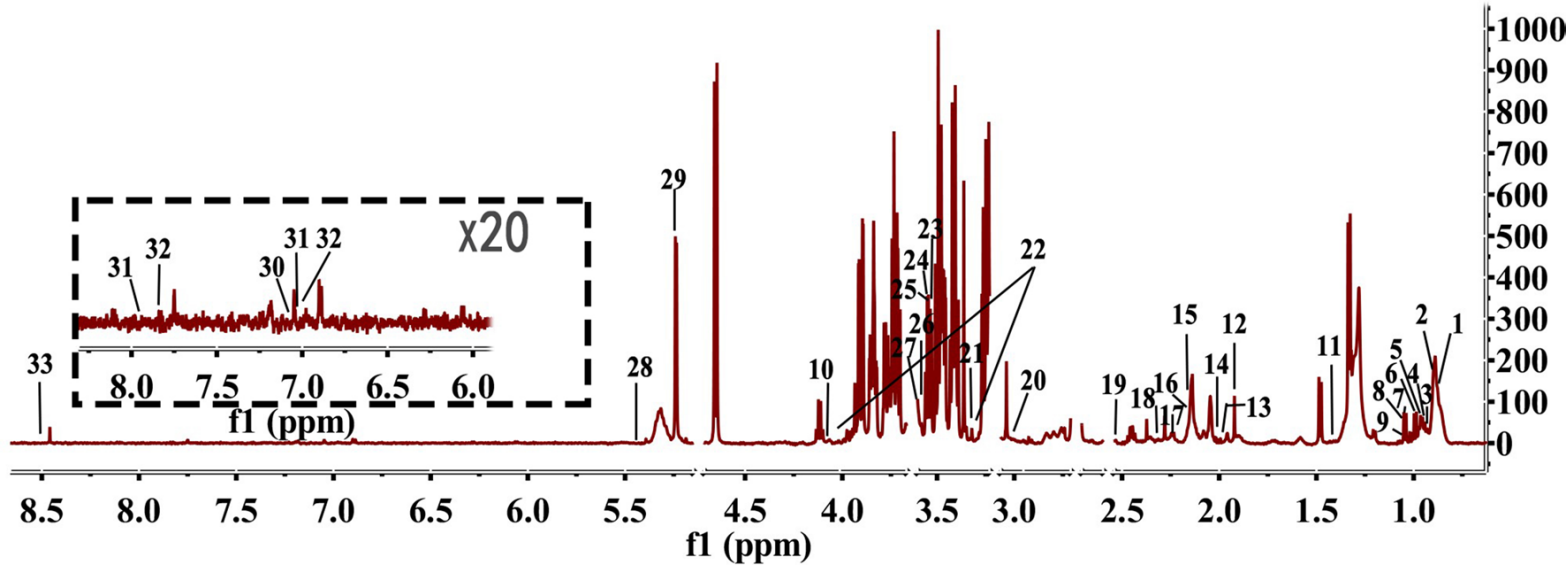
Typical ^1^H NMR spectra of extracts from serum (1, low-density lipoprotein; 2, very low-density lipoprotein; 3, isoleucine 4, leucine; 5, valine; 6, isobutyrate; 7, ethanol; 8, β-OH butyric acid esters; 9, methylmalonate; 10, lactic acid; 11, alanine; 12, acetic acid; 13, glutamic acid; 14, O-acetyl glycoprotein; 15, glutamine; 16, methionine; 17, formaldehyde; 18, acetylacetone; 19, citric acid; 20, creatinine; 21, phenylalanine; 22, choline; 23, choline phosphate; 24, glyceryl phosphate choline; 25, betaine; 26, glycine; 27, glycerol; 28, glycogen; 29, glucose; 30, tyrosine; 31, histidine; 32, methylhistidine; 33, toluene).

A typical nuclear magnetic resonance spectrum of serum (as shown in Fig. 4) is identified from the main metabolites of the spectrum according to published literature, the nuclear magnetic resonance database and the human metabolome database (HMDB: https://www.hmdb.ca/).

**Figure 5:**
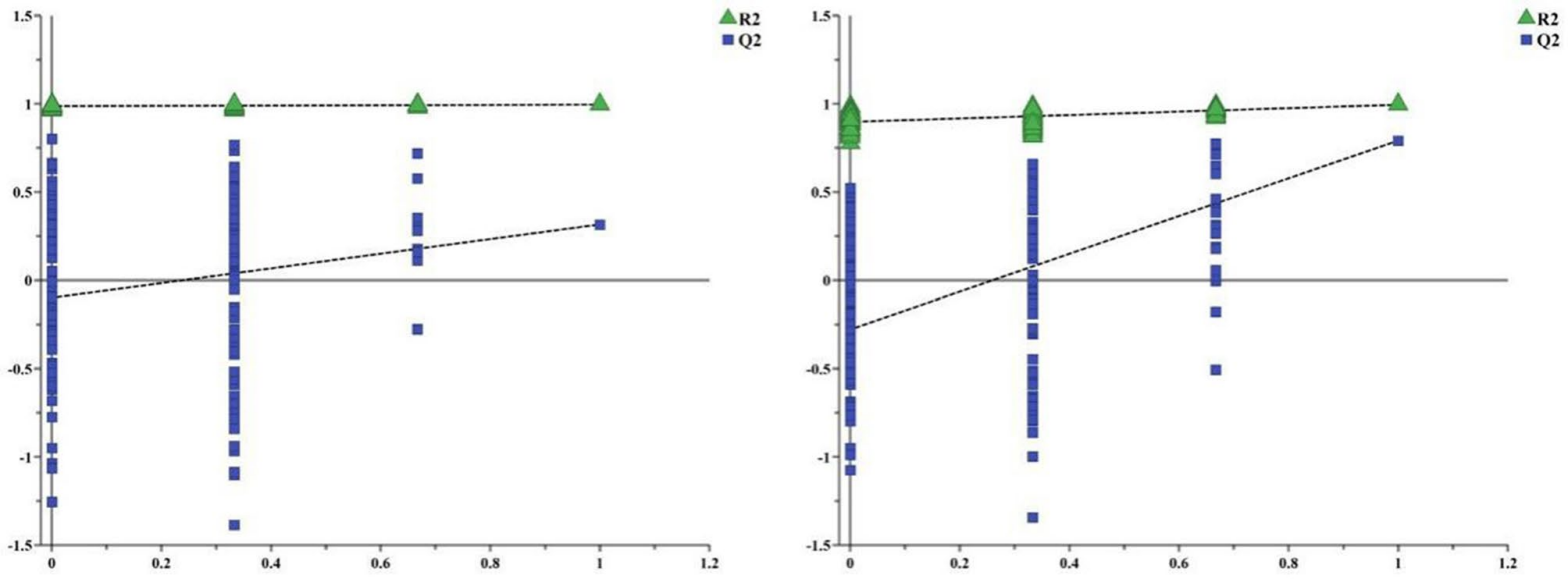
The model’s parameter height (R2) and prediction ability (Q2) are used to evaluate the quality of the OPLS-DA model. Legend: Theoretically, the closer the R2 and Q2 values are to 1, the better the accuracy of the model is, indicating that the model is more successful.

Statistical analysis was performed on all serum sample data matrices using PLS-DA. The results are shown in Fig. 5. The model group and the control group and the model group and electro-acupuncture group are distinctly separated.

**Figure 6:**
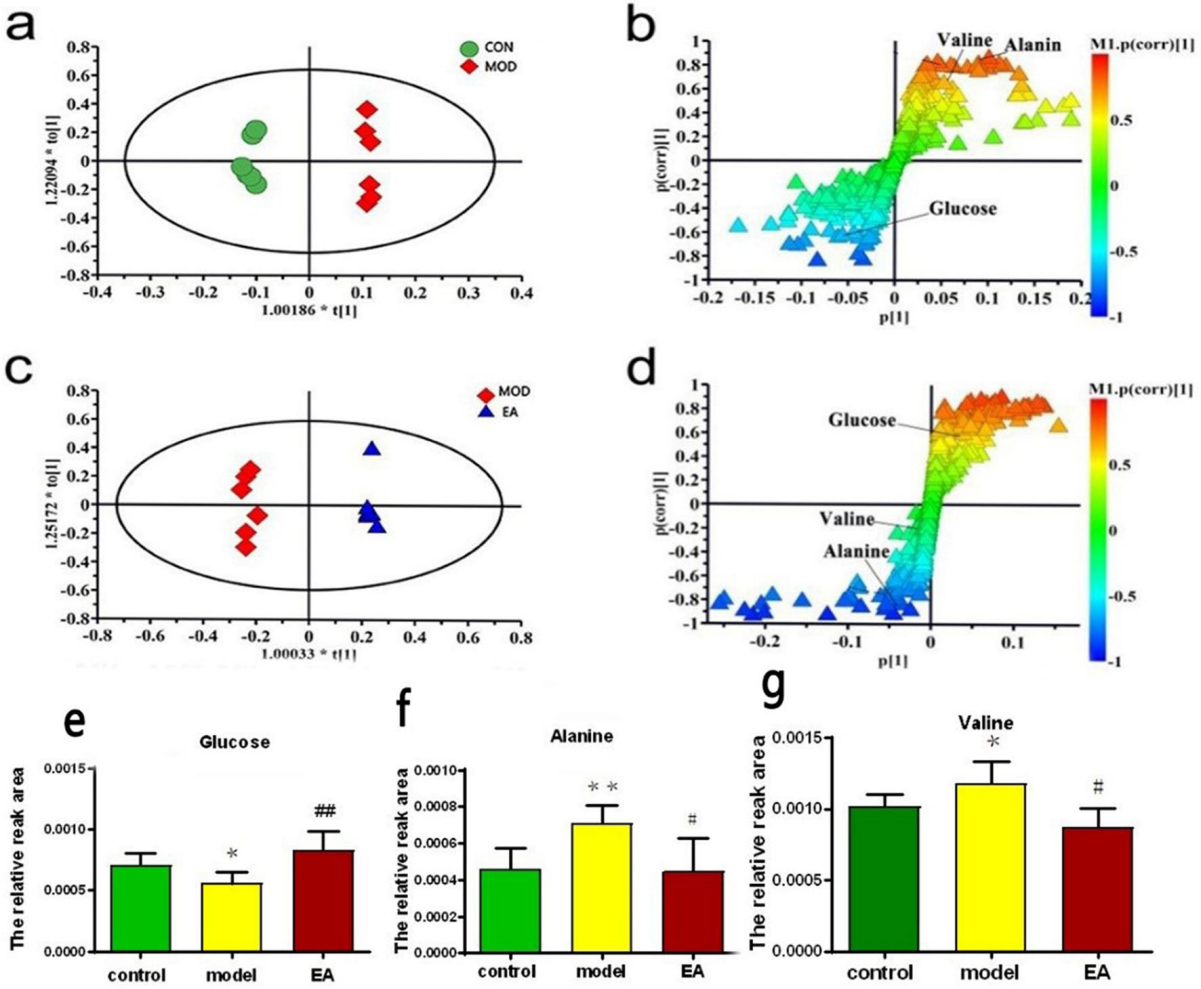
Distribution of S-plots corresponding to OPLS-DA maps in serum of each group. In (**b**) and (**d**) S-plot, the farther the VIP value on the “S” curve is from the origin, the greater the contribution to the packet. The control group and the model group [(**a**) and (**b**), R2X = 0.741, R2Y = 0.982, Q2 (cum) = 0.889] and the model group and the electro-acupuncture group serum [(**c**) and (**d**), R2X = 0.787, R2Y = 0.97, Q2 (cum) = 0.805]. For all sample types, a clear distinction is made between the control and model groups in (**a**). In (**c**), a good separation was observed between the model group and the electro-acupuncture group. Typical biomarker content [(**e**) glucose, (**f**) alanine, (**g**) valine) in the serum of each group of rats. In (**e**), (**f**) and (**g**), results are expressed as mean ± SE, n = 6 in each group. *p < 0.05, **p < 0.01 versus control group. #*p* < 0.05; ##*p* < 0.01 versus model group. Based on one-way analysis of variance, multiple comparisons were performed by LSD test.

To maximise the chances of endogenous metabolites in the serum of rats before and after modelling and the intervention of electro-acupuncture, the model group and the control group and the model group and the electro-acupuncture group were separately analysed by orthogonal partial least squares discriminant analysis (OPLS-DA) analysis. Figure 6 shows the OPLS-DA score map and S-plot map. In the S-plot, the farther the VIP value on the “S” curve is from the origin, the greater the contribution to the packet.

After screening for endogenous metabolites with VIP > 1 in this experiment, one-way analysis of variance was performed on the relative peak areas to which these metabolites belong, resulting in endogenous metabolites with significant differences in peak areas^18^. As can be seen from Fig. 6, the levels of alanine and valine in the model group were significantly higher than those in the control group (*p* < 0.05), and the glucose level was significantly decreased (*p* < 0.05). Compared with the model group, the serum alanine and valine in the EA group were decreased (*p* < 0.05), which was close to the control group, and the glucose levels were markedly increased (*p* < 0.05).

## Discussion

Long-term stress was found to have deleterious effects on the body’s physiological processes, leading to the body’s nervous, endocrine and immune disorders and other systemic diseases^3^. This study found that during chronic stress stimulation, rats’ body weight and thermal pain thresholds were significantly reduced. The test of thermal pain threshold has been used to measure hyperalgesia in various animal models^19^, and studies have found that mice under stress have a shorter thermal radiation latency^20^. This indicates the successful preparation of a chronic stress model. The zona glomerulosa of adrenal tissue become thinner, and the nucleus shrinks, which indicates that the chronic stress model will have adverse effects, which is consistent with the above view. However, these factors restored the normal state through EA at ST36, indicating that EA is very effective in improving chronic stress, but its treatment mechanism needs further explanation.

Some scholars believe that HPA axis abnormalities caused by various stresses play a vital role in chronic stress^21^. When the human body is stimulated by the external environment, the HPA axis becomes excited. CORT is an important stress hormone^22^ and glucose metabolism hormone in the human body and is a stable indicator for detecting the hypothalamic pituitary adrenal (HPA) axis response triggered by stress. In this study, we observed that under chronic stress conditions, CORT-secreted serum in the serum increased, and excessive CORT triggered negative feedback mechanisms of the hypothalamus, pituitary, and adrenals. This results in lower CRH levels in the hypothalamus and lower CRH levels in the serum. Eventually, the CORT of the adrenal gland decreases, leading to multiple dysfunctions of the system, such as rat weight, heat pain threshold, and adrenal pathological changes. This indicates that the model has been successfully prepared, which is consistent with the pathology of related scholars^23^. Electro-acupuncture ST36 can reduce the content of CORT in the serum, increase the content of CORT in the adrenal glands, increase the CRH content in the hypothalamus and serum, bring the body closer to normal levels and restore normal physiological functions. The effect of EA on chronic stress was shown to be closely related to the regulation of the HPA axis by electro-acupuncture. Evidence of similarity has been proposed by studies hypothesising that acupuncture can regulate the function of HPA by promoting CRH secretion in the hypothalamus, thereby regulating ACTH and CORT secretion^24^. These hormones are the main indicators of stress response through HPA^25^.

In this study, a nuclear magnetic metabolomics approach was used to study the interventional effects of EA on chronic stress models. We found that 3 serum metabolites, glucose, valine and alanine, are closely related to the occurrence of HIS and the role of electro-acupuncture. Glucose is synthesised from intermediates (such as acetic acid and glycerol) through a process called gluconeogenesis, which is usually glycolytic. Metabolic energy is captured during glycolysis. Glucose can oxidise and provide energy in the body. It is one of the most important substances in human metabolism. The decrease of serum glucose level in the model group, the abnormal glucose metabolism in the surface model rats, and the decrease of the thermal pain threshold in the model group may be related. Clinical symptoms of depression are similar. After electro-acupuncture intervention, the glucose in the serum showed a marked increase, which this indicates that needle intervention can significantly reverse glucose metabolism disorders in chronic stress model rats. Acupuncture has also been found to regulate glucose metabolism in the body^26^ which is consistent with this research.

Valine is called a branched chain amino acid because of its aliphatic side chain^27^, which is critical for human life, especially stress. In this study, an increase in valine levels in the model group compared to the control group indicates a shift in energy expenditure patterns to gluconeogenesis, and the formation of amino acid sugars may be the main pathway for amino acid metabolism. Compared with the model group, the decrease of valine in the electro-acupuncture group indicates that the amino acid regulation by electro-acupuncture may seriously affect its glucose metabolism. Valine reportedly regulates serum glucose levels and provides energy for physical activity^28^.

Alanine is transferred through the blood–brain carrier system related to the rate of 5-HT synthesis. Alanine levels in the serum of the model group rats increased, indicating that chronic stress leads to increased protein degradation. The positive significance of these changes is that they provide enough energy for the organism to respond to the “emergency situation”; however, the persistence of stress will lead to a decrease in body weight in rats. Due to negative nitrogen balance, lack of protein and decreased resistance of rats, the structure of adrenal tissue cells in the model group was changed, while electro-acupuncture caused the phenomenon of alanine levels in the serum of rats and tended to normal levels, indicating that electro-acupuncture ST36 improvement on chronic stress rats is related to regulating energy metabolism and negative nitrogen balance.

In summary, the effect of electro-acupuncture on glucose metabolism in chronic stress model rats is the main factor for its function.

Our research has limitations. First, we did not set up a sham acupuncture group. It is very important to set up a sham acupuncture group in the experiment. Some studies conducted a systematic review of the clinical trials using sham acupuncture control, and found that there was no difference in most of the treatment results between the sham acupuncture group and the acupuncture group, and there were doubts about the specificity of acupoints^29^. Other studies have shown that compared with the sham acupuncture group, the acupuncture group shows obvious advantages, and points out that the difference in clinical effect between the two groups is related to the specificity of acupoints^30–32^. Regretfully, we did not set up a sham acupuncture group because of the limited experimental conditions. But a sham acupuncture group will be definitely set up in future studies to verify our results. Second, this study used HPA axis-related hormone levels to validate the stress model. Studies have shown that stress can cause a range of effects, including gastric ulcers, increased adrenal mass, serum corticosterone levels, and plasma CK activity. Since the plasma samples used in the study were mainly used for the detection of ELISA and ^1^H NMR, the number of samples was limited, and other physiological indicators and the levels of enzymes in various pathways were not analysed^2, 33^. This will be carried out in subsequent studies. Finally, HPA axis is the main endocrine response of stress response , the hypothalamus is not only an important part of the HPA axis, but also the initial link of stress, changes in other brain regions (cerebral cortex, amygdala, hippocampus) under chronic stress conditions were not analysed.

In conclusion, a ^1^H NMR-based metabolomics analysis of serum samples coupled with pathological examination and ELISA examination was used to investigate effect of EA on glucose metabolism in chronic stress model rats. The results show that EA can improve the chronic stress state of rats by increasing the glucose level in the serum, reducing the levels of valine and alanine in the serum, regulating the body’s glucose metabolism.

Further research on electro-acupuncture requires intervention through gas chromatography-mass spectrometry (GC–MS) and liquid chromatography-mass spectrometry (LC–MS) metabolomics to validate our findings. The current work shows that the effect of electro-acupuncture on chronic stress is likely to be achieved by regulating glucose metabolism, which can provide a reference for clinical acupuncture treatment of depression.

## Methods

### Experimental instruments and software

0.30 × 25 mm stainless steel acupuncture needle (Suzhou Acupuncture Supplies Co., Ltd., Jiangsu, China); IITC-336G foot thermal pain tester (Shanghai Yuyan Scientific Instrument Co., Ltd.); LH202 Han’s acupoint neurostimulator (produced by Peking University Neuroscience Research Institute, produced by Beijing Huawei Industrial Development Co., Ltd.); Bruker 600 MHz Advanced III NMR spectrometer (Brook, Germany). Germany LEICA, Intelligent Biomicroscope (Olympus); BX53.Leica paraffin embedding station (EG 1,160, Leica Biosystems Nussloch GmbH, Germany); Leica rotary microtome (RM 2,135, Leica Biosystems Nussloch GmbH, Germany); TSPd4 (Cambridge Isotope Laboratories, Inc., USA); K2HPO4, NaH2PO4⋅2H2O (Xilong Scientific Co., Ltd., Guangdong, China); D2O (633,178, Sigma, USA); anhydrous ethanol, 0.9% sodium chloride injection, 4% polyoxymethylene (Changsha GugeBio Technology Co., Ltd., Changsha, China); NMR spectrometer (Bruker Biospin, Rheinstetten, Germany); NMR data preprocessing (MestReNova v9.0.1 software, Mestrelab Research, Santiago de Compostela, Spain); multivariate statistical analysis (SIMCA-P14.1, Umetrics, Sweden); R510 small animal anaesthesia machine (Rived R510IP).

### Animal treatment

A total of 75 clean male Sprague–Dawley rats were used in the study [License No. (Beijing) 2014-0013], which purchased from the Chinese Food and Drug Control Institute. The rats were acclimated for at least 7 days and maintained with a 12-h light/dark cycle with free access to water and food. Five rats were placed in each cage in a cleanroom. The rats were housed in a controlled environment (20 ± 1)°C. All rats were randomly divided into 3 groups (n = 25 in each group): control group, model group and electro-acupuncture group. Each group of rats was free to drink clean water and a standard rat diet. At 9:00 in the morning, the rats in the model group and EA group were prepared with HIS. At 3 pm, the rats in the EA group were stimulated by EA for 30 min. The rats in the control group and the model group were bound with 30 min with a rat retainer (Chinese patent application number: 201110021482.5, State intellectual property Office) during EA.

HIS involves subjecting Sprague–Dawley rats to 3 different types of stressors, each stressor in an unpredictable manner for 9 days. Every day at 9 a.m., rats in the model group and electro-acupuncture group were prepared with HIS. The stimulation method included 4 °C cryopreservation pressure (CRS), at 45 min per group. Rats were subjected to 60 min of water stress (WAS) and 20 min of forced swimming stress (FSS)^34^. At baseline and 9 days later, the rats in each group were tested for body weight and thermal radiation latency, then anesthesia was induced with 4% isoflurane at 0.8 L air flow, then maintained with 1.5% isoflurane.

### Electro-acupuncture treatment

At 3 p.m. every day, rats in the electro-acupuncture group received electro-acupuncture stimulation on bilateral ST36 for 30 min. ST36 positioning: approximately 5 mm below the tibial tubercle of the knee joint. According to “The Veterinary Acupuncture of China” and Government Channel and Points Standard GB12346-90 of China. We used stainless steel needles with a diameter of 0.25 mm and a length of 30 mm (Suzhou Huatuo Medical Devices Co., Ltd.). The needle was inserted 2 mm into the skin. Han’s acupoint nerve stimulator (Beijing LH202 type) was connected to the double-sided ST36, dense wave (100 Hz is 1.05 s), 2 Hz alternates 2.85 μs, and pulse width is 0.1 ms). The electrical stimulation intensity was set to the observable muscle twitch (approximately 1 mA) and stimulated for 30 min a day for a total of 9 days.

### Indicator observation and detection

#### Rat body weight and behavioural testing

Rat weight and behavioural tests were performed before (baseline) and after treatment. Rats were fasted for 6 h before testing. They were then placed in the transparent compartment of the foot thermal pain tester for 10 min. When they were quiet, the heel was heated, and the rat’s lifting time was observed. This was repeated 7 times, and the average was selected to record the results^35^.

#### Adrenal tissue pathology

The adrenal tissue from 3 groups of rats was collected and fixed in 10% formaldehyde. After dehydration with gradient ethanol, the tissue examination was embedded, sliced at 6 m with an Ultra-Tin semi-automatic slicer and dyed with haematoxylin and eosin. Adrenal cortical cells were observed under light microscopy after HE staining. Finally, computer processing was performed using the Motictek 3.1 image acquisition system.

#### Enzyme linked immunosorbent assay (ELISA) assessment

All rats were decapitated to collect 5 mL of blood, and serum samples were obtained by centrifugation (10,000 rmp, 10 min, 4 °C). Each serum sample was aliquoted and stored in a − 80 °C refrigerator for ELISA and ^1^H-NMR experiments. Adrenal and hypothalamic tissues were quickly excised on ice, fixed with liquid nitrogen, and stored in a − 80 °C refrigerator. The samples of adrenal gland, hypothalamus and plasma were taken out from the refrigerator at − 80 °C and placed on ice at a temperature of 2–4 °C for melting. A certain amount of PBS solution (PH7.4) was added to the cryopreservation tube of the specimens of each group. After the tissue was fully homogenized by a homogenizer, it is placed on a centrifuge at 4 °C, 3,000 revolutions per minute, and centrifuged for 20 min. Collect the supernatant carefully and put it in the new EP tube for testing. Determination of CORT and CRH levels in serum, adrenal glands and hypothalamus using an ELISA kit purchased from R&D Systems (Minneapolis, USA) and Phoenix Pharmaceuticals (California, USA). The ELISA-based method was performed according to the instructions provided by the manufacturer.

#### ^1^H NMR experiments

The serum samples tested by ^1^H-NMR were taken out of the − 80 °C refrigerator and placed on ice until the plasma samples melted. The hydrogen nuclear magnetic resonance detection of serum samples was carried out according to the method provided by the manufacturer^36^: 450 μL serum and 350 μL phosphate buffer (90 mM K2HPO4/NaH2PO4, pH 7.4, 99.9% D2O) were shaken well, centrifuged at 4 °C at a speed of 10,000 rpm for 20 min, and the supernatant of 500 μL was transferred to the nuclear magnetic resonance tube of 5 mm for for NMR experiment. The ^1^HNMR spectra of serum samples were obtained by 600 MHz Bruker spectrometer at 298 k, and the ^1^HNMR spectra were collected by standard one-dimensional Carr-Purcell-Merboom-Gill (CPMG, RD-90 (TCP-180-TCP)-Acquisition) water suppression. CPMG sequence can suppress the signals of water peaks and macromolecular substances to detect small molecular metabolites in serum. The specific settings are as follows: the scanning times is 64, the spectral width is 12.019 kHz, the relaxation time is 320 ms. The other parameters are set as follows: PW = 30 °C (12.7 μs), RD = 1.0) for LB = 0.3 Hz conversion.

#### ^1^H-NMR spectrogram pretreatment and data analysis

The nuclear magnetic spectrum was processed using MestReNova (version 8.0.1, Mestrelab Research, Santiago de Compostella, Spain). According to the literature method^37^, the spectrum was corrected by the chemical shift of the creatinine methyl peak (δ3.04) as the standard, and the phase and baseline adjustment were performed to remove the water peak (δ4.5 to 5.2) interference. The displacement interval (δ = 4.75–5.00) is segmented and integrated according to δ = 0.01 steps, and the obtained integral value is normalised and then imported into the SIMCA = P 13.0 software (Umetric, Sweden). Using Pareto conversion (Pareto Scaling), it was subjected to pre-treatment and principal component analysis (PCA) for preliminary analysis, followed by partial least squares discriminant analysis (PCA-DA)^38,39^. Additionally, parameters for model fitness (R2) and predictive ability (Q2) were used to assess quality of OPLS-DA model. And the *p* value from cross-validated analysis of variance (CV-ANOVA) was also calculated to indicate the level of significance for group separation in OPLS-DA. Finally, discover the potential variables for differentiation, the corresponding S-plot of OPLS-DA model was conducted. The potential biomarkers were extracted according to the variable importance in the project (VIP) of the established OPLS-DA model (VIP ≥ 1.00) and an independent-sample *t *test (*p* < 0.05) using (OriginProver.8.1).We score the plot and corresponding S-plot and find endogenous differential metabolites^14^. The data are processed by SPSS20.0 software, and the measured data are expressed by mean ± standard deviation (x ± s). Through analysis-nonparametric test-old dialog box-sample Kmurs. The *p* value was detected and compared, it was found that *p* > 0.05, which showed that there was no significant difference between *p* value and normal distribution, and the data showed normal distribution. All the data obeyed normal distribution, and the data with the same variance were analyzed by one-way analysis of variance (ANOVA), and further pairwise comparison was performed by least significant difference (LSD) test. All *p* values were 2 tailed with significance level at 0.05.

### Ethics statement

Animal care and experimental procedures used in this study have been obtained Animal Care and Use Committee of Xiamen University (Permit Number: SCXK 2014-0001). The study was based on the care and use of laboratory animals at the National Institutes of Health.
